# DISSECT Method Using PNA-LNA Clamp Improves Detection of *EGFR* T790m Mutation

**DOI:** 10.1371/journal.pone.0067782

**Published:** 2013-06-21

**Authors:** Minakshi Guha, Elena Castellanos-Rizaldos, G. Mike Makrigiorgos

**Affiliations:** 1 Division of DNA Repair and Genome Stability, Department of Radiation Oncology, Dana-Farber Cancer Institute, Harvard Medical School, Boston, Massachusetts, United States of America; 2 Division of Medical Physics and Biophysics, Department of Radiation Oncology, Dana-Farber Cancer Institute, Brigham and Women's Hospital, Harvard Medical School, Boston, Massachusetts, United States of America; UT MD Anderson Cancer Center, United States of America

## Abstract

Non-small cell lung cancer (NSCLC) patients treated with small molecule *EGFR* inhibitors, such as gefitinib, frequently develop drug resistance due to the presence of secondary mutations like the T790M mutation on *EGFR* exon 20. These mutations may originate from small subclonal populations in the primary tumor that become dominant later on during treatment. In order to detect these low-level DNA variations in the primary tumor or to monitor their progress in plasma, it is important to apply reliable and sensitive mutation detection methods. Here, we combine two recently developed methodologies, Differential Strand Separation at Critical Temperature (DISSECT), with peptide nucleic acid-locked nucleic acid (PNA-LNA) polymerase chain reaction (PCR) for the detection of T790M *EGFR* mutation. DISSECT pre-enriches low-abundance T790M *EGFR* mutations from target DNA prior to implementing PNA-LNA PCR, a method that can detect 1 mutant allele in a background of 100–1000 wild type alleles. The combination of DISSECT and PNA-LNA PCR enables the detection of 1 mutant allele in a background of 10,000 wild type alleles. The combined DISSECT-PNA-LNA PCR methodology is amenable to adaptation for the sensitive detection of additional emerging resistance mutations in cancer.

## Introduction

Mutations in the epidermal growth factor receptor (*EGFR*) are found in many advanced lung cancer patients who respond positively to *EGFR* tyrosine kinase inhibitors, gefitinib or erlotinib [1], [2]. However, many of these patients eventually relapse and develop resistance to these *EGFR* inhibitors. The T790M mutation on exon 20 of the *EGFR* receptor has been reported as one of the driving mutations for the acquired resistance to gefitinib treatment [3]. Approximately half of the patients that acquire resistance to gefitinib are found to harbor the T790M *EGFR* mutation [3], [4]. Many of the patients that do relapse often harbor pre-existing T790M *EGFR* mutation at very low levels within the original tumor population, leading to resistance after gefitinib treatment [5].

Screening patients for low level T790M *EGFR* mutations prior to administering gefitinib treatment may be useful for assessing the possibility of disease relapse. Alternatively, monitoring of T790M mutations in plasma during EGFR treatment may be useful for future clinical decision making [6]. In both cases, detection of mutated DNA is masked by an exceedingly high amount of wild type DNA, which is a common technical problem when examining surgical biopsies or samples obtained from bodily fluids such as plasma or sputum [7]–[9]. The peptide nucleic acid-locked nucleic acid (PNA-LNA) real time polymerase chain reaction is a rapid and sensitive method that detects *EGFR* mutations in the presence of 100- to 1000-fold wild type background [10]. This method has recently been adapted for the detection of T790M *EGFR* mutation in gefitinib-refractory disease by the use of the PNA “clamp” to inhibit the amplification of wild type DNA [11], [12]. However, there is a need to improve the detection of the T790M mutation even further than 1∶1000, as the mutation may be present in a very small population of tumor cells [13]. We recently developed a novel method based on Differential Strand Separation at Critical Temperature (DISSECT) to enrich for low level mutations in DNA samples using magnetic bead-conjugated DNA probes [14]. DISSECT utilizes the differential denaturation properties of DNA heteroduplexes and can therefore enrich mutations at any position on the sequence, enabling mutation scanning and discovery [14]. Since the target sequence remains unmodified during DISSECT, the resulting mutation-enriched DNA fraction can be used as a template for any existing downstream detection method, including PNA-LNA PCR.

Here we demonstrate the novel combination of DISSECT and PNA-LNA PCR to identify extremely low levels of T790M *EGFR* mutation. We demonstrate that a preliminary step of mutant enrichment using DISSECT results in a major improvement of PNA-LNA PCR detection method and increases the sensitivity of detection to at least 1 mutant allele in a background of 10,000 wild type alleles.

## Materials and Methods

### Cell Lines and Genomic DNA isolation

Human male genomic DNA (Promega, cat. No. G1471) was used as wild type control for dilution experiments with DNA containing the T790M mutation. Genomic DNA from mutated cell line H1975 (ATCC no. CRL-5908™) was extracted using the DNeasy Blood and Tissue Kit according to manufacturer's protocol (Qiagen). DNA concentrations for both mutant and wild type DNA were determined by using the Nanodrop 1000 spectrophotometer (Thermo Scientific).

### PCR pre-amplification and High Resolution Melt Analysis

We first amplified 20 ng of both wild type male and 10% H1975 genomic DNA using conventional PCR in a 25 µl reaction in the presence of DNA intercalating dye, 1×LCGreen (Idaho Technology). Briefly, PCR reagents were: 1×GoTaq Flexi Buffer (Promega), 2.5 mmol/L MgCl_2_, 0.2 mmol/L of each dNTP, 0.63U of GoTaq Flexi DNA polymerase (Promega) and 0.2 µmol/L of forward (5′-GCTGGGCATCTGCCTCACCTCCACCGTGCA**A**CT-3′) and reverse primer (5′-GTCTTTGTGTTCCCGGACATAG-3′). Forward primer was designed to replace the A/G SNP present in *EGFR* exon 20 in order to generate a uniform template [15]. Thermal cycling was done on the Cepheid SmartCycler™ using the following conditions: initial denaturation at 95°C for 2 min, followed by 20 cycles at 95°C for 15 s, 55°C for 30 s and 72°C for 30 s. Serial mutant dilutions (1%, 0.1%, and 0.01%) were obtained by mixing pre-amplified wild type and 10% mutant DNA to ensure that enough number of mutant copies were present in the lower mutant dilution samples. Prior to further analysis, mutant fractions were validated by PCR in the presence of 1×LCGreen followed by High Resolution Melt (HRM) analysis as reported previously [16].

Alternatively, to apply the DISSECT-PNA-LNA PCR approach directly from mixed genomic DNA (as opposed to mixing PCR products from mutant and WT) we used Phusion Hi-Fidelity™ DNA polymerase (New England Biolabs, Ipswich, MA) for the pre-amplification from 300 ng of genomic wild type DNA and H1975 mutant DNA (10%, 1%, 0.1%, and 0.01% mixtures). The lowest dilution was anticipated to contain approximately 10 mutant molecules in the reaction. Pre-amplification was done in 25 µl reaction volume for a total of 20 cycles according to manufacturer's protocol (New England Biolabs). For this reaction, 0.3 µmol/L of the forward primer 5′-TGGGCATCTGCCTCACCT-3′ was used together with the reverse primer 5′-GTCTTTGTGTTCCCGGACATAG-3′.

### DISSECT protocol and probe design

Streptavidin-coated Dynabeads (Life Technologies, Grand Island, NY) were coupled to dual-biotinylated probe sequence of 35 nt long complementary to the wild type allele of *EGFR* exon 20 (5′-Dual-biotin-Spacer 18-CCA GGA GGC AGC CGA AGG GCA TGA GCT GCG TGA TG-3′; synthesized by Integrated DNA Technologies Inc., Coralville, IA, USA). This probe was then conjugated to streptavidin beads as per manufacturer's protocol (Invitrogen Life Technologies). These Dynabead-probes were finally resuspended in a stock buffer containing 6X SSPE (0.9 M NaCl, 60 mM Na_2_HPO_4_, 6 mM EDTA) to a final concentration of 10 µg/µL and kept at 4°C.

About 10 µL of Dynabead-probes were incubated with a 1∶50 dilution of pre-amplified DNA in a total volume of 50 µl of 1×Phusion Buffer. A ‘step-down anneal’ protocol was used to hybridize DNA onto beads by denaturing the DNA-bead solution at 95°C for 2 min, then annealing at 60°C for 2 min, 58°C for 2 min, 56°C for 2 min, 54°C for 1 min and 25°C for 5 min using an Eppendorf Mastercycler EP machine (Eppendorf Inc., Hauppauge, NY). Two repeated cycles of *EGFR* mutant denaturation from beads were done according to DISSECT protocol as described previously [14]. Critical denaturation temperature for T790M mutant *EGFR* DNA was achieved by heating the bead solution at a temperature of 76°C for 2 min followed by magnetization, and collection of supernatant enriched for mutant *EGFR* ssDNA sequences. Critical temperature determination was done as described previously [14]. 5 µL of DNA eluted from beads following two rounds of DISSECT was amplified by PNA-LNA PCR protocol [10], [11] and analyzed by Sanger Sequencing (Eton Bioscience, Cambridge, MA) using sequencing primer 5′-40T-GCTGGGCATCTGCCTCA-3′. Prior to sequencing, PCR products were treated with Exonuclease I (New England Biolabs) and shrimp alkaline phosphatase (USB/Affymetrix).

### Detection of T790M mutation by PNA-LNA PCR clamp assay

The PNA-LNA clamp assay was done as reported previously [11]. Briefly, reagent concentrations were the same as reported in the PCR pre-amplification step, but 1×LCGreen was replaced by 0.2 µmol/L of the PNA clamp (NH_2_-CTCATCA**C**GCAGCTCA-COOH), complementary to the wild type allele, and 0.2 µmol/L of the fluorescent-labeled mutation detection probe containing a locked nucleic acid (indicated by a plus sign), 6-FAM/ CTCATCA+**T**GCAGCTCATG/BlackHoleQuencher-1 [11]. Cycling conditions for PNA-LNA PCR were as following: 95°C for 2 min, followed by 70 cycles at 95°C for 15 s, and 60°C for 30 s.

## Results and Discussion

### Principle of DISSECT and PNA-LNA PCR

**Figure 1:**
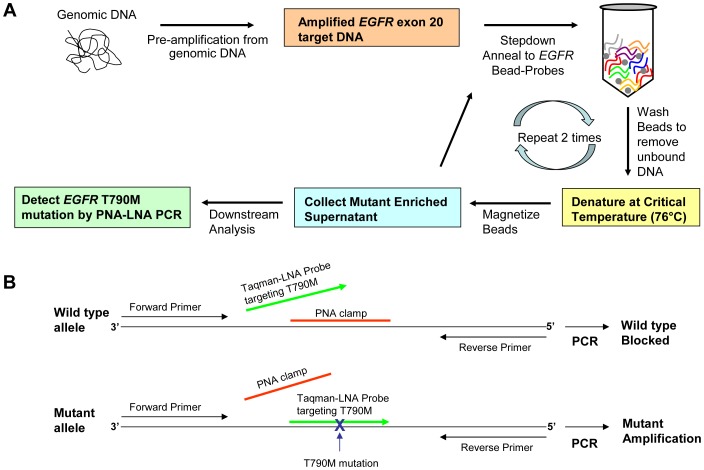
Schematic of procedure. (A) Flowchart diagram of *EGFR* exon 20 mutant enrichment by DISSECT followed by PNA-LNA PCR. (B) Schematic design of *EGFR* exon 20 primers, LNA probe, and PNA clamp during PNA-LNA PCR procedure.

The flow diagram of the procedure presented in **Figure 1A** demonstrates a method for the enrichment of low level *EGFR* mutations using a combination of DISSECT protocol followed by PNA-LNA PCR. Pre-amplification from genomic DNA using gene-specific primers allows the generation of target DNA (i.e. *EGFR* exon 20) that can subsequently be captured on to magnetic beads coated with sequence-specific wild type probes. In this step-down anneal protocol, double-stranded target DNA is denatured at 95°C followed by a gradual decrease in temperature where single-stranded DNA anneals to complementary probe sequences immobilized on magnetic beads. These beads are then washed to eliminate the unbound DNA and then subjected to a differential denaturation step. For differential denaturation, the temperature is raised to a critical temperature (T_c_) of the probe sequence (i.e. 76°C) where the mutation-containing target DNA separates preferentially from the beads, resulting in a supernatant enriched with the mutant DNA fraction [14]. DISSECT is repeated twice prior to collecting the final supernatant and applying downstream analysis. We used the PNA-LNA PCR as a downstream analysis method to detect T790M *EGFR* mutation as described previously [11]. In **Figure 1B**, a schematic representation of the PNA-clamp is shown, along with primers and TaqMan LNA probe for real time detection of the T790M *EGFR* mutation. By combining the two procedures (DISSECT plus PNA-LNA PCR) we were able to identify mutation abundances of 0.01% as compared to PNA-LNA PCR alone, where only 0.1% mutation abundances could be reliably distinguished.

### Sensitivity of Detection of T790M*EGFR* mutation

**Figure 2:**
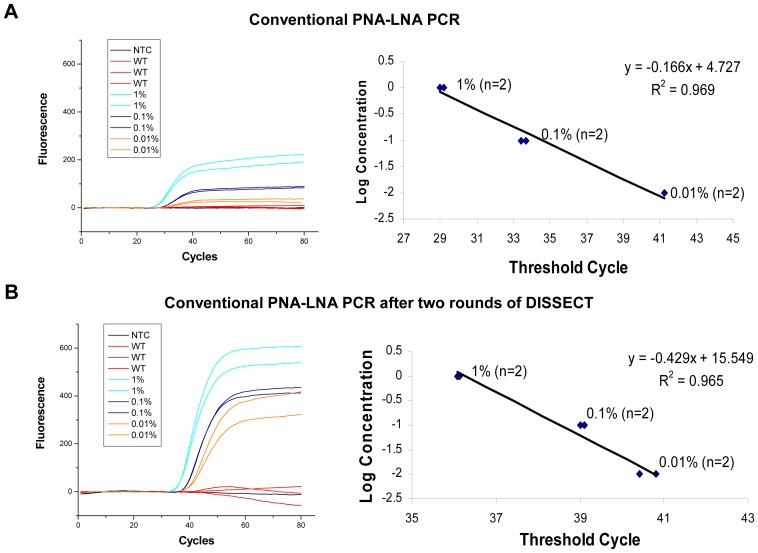
Comparison of PNA-LNA sensitivity of detection for the T790M*EGFR* mutation. (A) Real-time PCR plot represents serially diluted PCR product with T790M mutation (1%, 0.1% and 0.01%) into amplified product from wild type DNA, by conventional PNA-LNA PCR. (A, right) Corresponding plot of the log concentration of T790M mutant DNA versus threshold cycle number. Graph shows mutant T790M serial dilution following conventional PNA-LNA PCR. (B) Real-time PCR plot shows an increased level of detection when PNA-LNA is applied to a sample that has undergone two rounds of mutant enrichment by DISSECT. (B, right) Corresponding graph shows mutant T790M serial dilution following PNA-LNA PCR after DISSECT.

To obtain a panel of T790M *EGFR* mutations, we made serial dilutions of pre-amplified PCR product from H1975 lung cancer cell line, which contains the T790M mutation, into PCR product from wild type human male genomic DNA. A dilution gradient from 1% to 0.01% mutant-to-wild type DNA was generated to evaluate the sensitivity of detection of PNA-LNA PCR. In **Figure 2A**, PNA-LNA real-time PCR could reliably detect 1 mutant copy in a background of 1000 wild type copies of DNA. The corresponding plot shows the log concentration of T790M mutant DNA versus threshold cycle number and reveals an R-squared value of 0.969 for mutant T790M serial dilution following conventional PNA-LNA PCR. To improve the sensitivity of detection, we added a preliminary step of DISSECT-based mutant enrichment followed by PNA-LNA PCR. In **Figure 2B**, we show that DISSECT improves PNA-LNA PCR detection of T790M *EGFR* mutation by at least one order of magnitude and clearly identifies an initial 0.01% mutant dilution when conventional PNA-LNA PCR is preceded by two rounds of DISSECT. Plotting of the log concentration of T790M mutation versus threshold cycle number results in an R-squared value of 0.965. Since no polymerase synthesis is employed during the mutant enrichment step, DISSECT allows for a quantitative pre-enrichment of the *EGFR* gene mutant population minimizing the introduction of polymerase-induced errors. This method is optimal prior to PNA-LNA PCR as it does not require enzymatic manipulation of the starting material, resulting in an unmodified sequence for downstream analysis protocols such as PNA-LNA PCR.

As an additional verification we applied the method to 300 ng of genomic DNA starting material, instead of mixing PCR products. This was done to ensure that at least one of the two systems (PCR or genomic DNA mixtures) had rigorously measured proportions of tumor and normal DNA. The serial dilutions formed were from 10% to 0. 01% H1975 mutant genomic DNA into wild type male genomic DNA. An initial pre-amplification was performed as described in the methods using Phusion Hi-Fidelity™ DNA polymerase. The results were similar to mixing PCR products as mentioned above, such that PNA-LNA real-time PCR could detect down to ∼0.1%, while two rounds of DISSECT preceding PNA-LNA PCR could identify an initial mutation abundance of 0.01% (**Figure S1**).

### Analysis of T790M*EGFR* mutant enrichment by Sanger Sequencing

**Figure 3:**
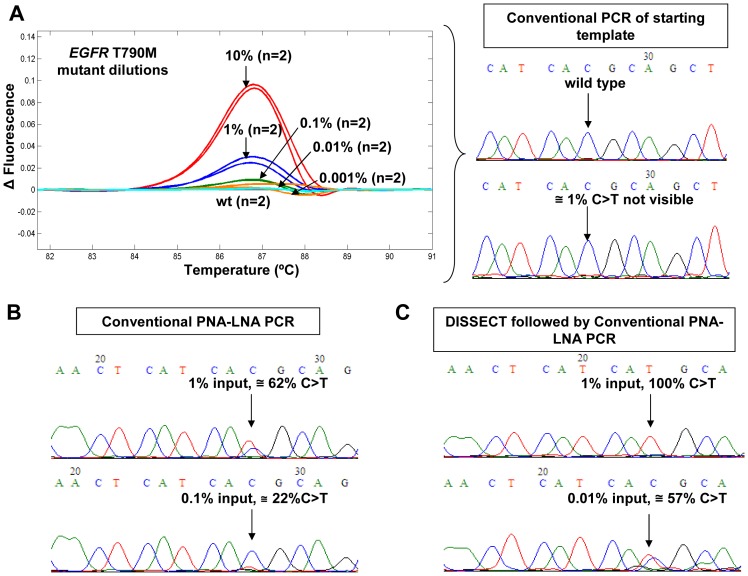
T790M*EGFR* mutant enrichment analysis by HRM and Sanger Sequencing. (A) HRM analysis plots and Sanger Sequencing chromatograms of serial dilutions of T790M *EGFR* mutations versus wild type DNA following conventional PCR. (B) The detection after mutant enrichment by conventional PNA-LNA PCR using an initial mutant abundance of 1% and 0.1% T790M, c.2369C>T. (C) Improved detection after mutant enrichment from two rounds of DISSECT followed by conventional PNA-LNA PCR from an initial mutant abundance of 1% and 0.01% T790M, c.2369C>T.

To further validate the combination of DISSECT and PNA-LNA PCR method, we analyzed PNA-LNA PCR amplicons using HRM and Sanger Sequencing. In **Figure 3A**, we used pre-amplified DNA as starting template and applied a nested conventional PCR to all serial dilutions followed by HRM and direct sequencing. As expected, this approach did not confer any selectivity towards mutant sequences since HRM detected between 1% and 10% mutation abundance, while Sanger sequencing chromatograms were unable to identify the presence of 10% or lower T790M mutation abundance. When PNA-LNA PCR was implemented instead of conventional PCR, a mutant enrichment of ∼60-fold resulted from an initial input mutant abundance of 1% T790M, c.2369C>T as shown in **Figure 3B**. Sanger sequencing after conventional PNA-LNA PCR identified an initial mutation abundance of 0.1–1%, in agreement with Miyazawa et al [11]. When both DISSECT and PNA-LNA PCR were combined in **Figure 3C**, the detection by Sanger sequencing was improved to at least ∼0.01% mutation abundance, i.e. an improvement of one order of magnitude.

The combination method described above is an improvement over PNA-LNA PCR approaches developed previously [17] as this protocol is significantly shorter and it does not require a high amount of starting DNA. Additionally, the present approach provides a greater discrimination for the detection of the T790M mutation compared to our previous report where two consecutive rounds of COLD-PCR combined with TaqMan were necessary to detect 0.05% mutant abundances [18]. We anticipate that DISSECT-PNA-LNA PCR will prove useful in detecting low-level mutations in tumor tissue prior to, or after administering gefitinib treatment to NSCLC patients. Moreover, the combined method can be used to detect other mutations, such as those identified in *KRAS* via FLAG (Fluorescent Amplicon Generation) or CO-amplification at Lower Denaturation temperature-PCR (COLD-PCR) [18]–[22], or other *EGFR* mutations in NSCLC (ie. L858R *EGFR* mutation) to help improve lung cancer diagnosis and follow-up treatment options.

## Supporting Information

Figure S1 **PNA-LNA PCR sensitivity of detection of the T790M mutation when starting from genomic DNA using Phusion Hi-Fidelity™ DNA polymerase.** (A) Conventional PNA-LNA PCR showing the detection of the T790M mutant dilutions of 10%, 1%, 0.1% and 0.01% mutant-to-wild type ratios versus wild type alone. (A, right) Corresponding plot of the log concentration of T790M mutant DNA versus threshold cycle number. (B) Real-time PCR plot showing the detection for 1%, 0.1% and 0.01% mutant-to-wild type ratios when PNA-LNA is applied to a sample that has undergone two rounds of mutant enrichment by DISSECT. (B, right) Corresponding graph shows mutant T790M serial dilution following PNA-LNA PCR after DISSECT.
(TIF)

## References

[1] Perez-Soler R, Chachoua A, Hammond LA, Rowinsky EK, Huberman M, et al (2004) Determinants of tumor response and survival with erlotinib in patients with non–small-cell lung cancer. J Clin Oncol 22: 3238–3247. 15310767 10.1200/JCO.2004.11.057

[2] Paez JG, Janne PA, Lee JC, Tracy S, Greulich H, et al (2004) EGFR mutations in lung cancer: correlation with clinical response to gefitinib therapy. Science 304: 1497–1500. 15118125 10.1126/science.1099314

[3] Kobayashi S, Boggon TJ, Dayaram T, Janne PA, Kocher O, et al (2005) EGFR mutation and resistance of non-small-cell lung cancer to gefitinib. N Engl J Med 352: 786–792. 15728811 10.1056/NEJMoa044238

[4] Kosaka T, Yatabe Y, Endoh H, Yoshida K, Hida T, et al (2006) Analysis of epidermal growth factor receptor gene mutation in patients with non-small cell lung cancer and acquired resistance to gefitinib. Clin Cancer Res 12: 5764–5769. 17020982 10.1158/1078-0432.CCR-06-0714

[5] Pao W, Miller VA, Politi KA, Riely GJ, Somwar R, et al (2005) Acquired resistance of lung adenocarcinomas to gefitinib or erlotinib is associated with a second mutation in the EGFR kinase domain. PLoS Med 2: e73. 15737014 10.1371/journal.pmed.0020073 PMC549606

[6] Kuang Y, Rogers A, Yeap BY, Wang L, Makrigiorgos M, et al (2009) Noninvasive detection of EGFR T790M in gefitinib or erlotinib resistant non-small cell lung cancer. Clin Cancer Res 15: 2630–2636. 19351754 10.1158/1078-0432.CCR-08-2592 PMC2727796

[7] Milbury CA, Li J, Makrigiorgos GM (2009) PCR-based methods for the enrichment of minority alleles and mutations. Clin Chem 55: 632–640. 19201784 10.1373/clinchem.2008.113035 PMC2811432

[8] Sidransky D, Von Eschenbach A, Tsai YC, Jones P, Summerhayes I, et al (1991) Identification of p53 gene mutations in bladder cancers and urine samples. Science 252: 706–709. 2024123 10.1126/science.2024123

[9] Tada M, Omata M, Kawai S, Saisho H, Ohto M, et al (1993) Detection of ras gene mutations in pancreatic juice and peripheral blood of patients with pancreatic adenocarcinoma. Cancer Res 53: 2472–2474. 8495407

[10] Nagai Y, Miyazawa H, Huqun, Tanaka T, Udagawa K, et al (2005) Genetic heterogeneity of the epidermal growth factor receptor in non-small cell lung cancer cell lines revealed by a rapid and sensitive detection system, the peptide nucleic acid-locked nucleic acid PCR clamp. Cancer Res 65: 7276–7282. 16105816 10.1158/0008-5472.CAN-05-0331

[11] Miyazawa H, Tanaka T, Nagai Y, Matsuoka M, Sutani A, et al (2008) Peptide nucleic acid-locked nucleic acid polymerase chain reaction clamp-based detection test for gefitinib-refractory T790M epidermal growth factor receptor mutation. Cancer Sci 99: 595–600. 18271876 10.1111/j.1349-7006.2007.00706.x PMC11158388

[12] Nielsen PE, Egholm M (1999) An introduction to peptide nucleic acid. Curr Issues Mol Biol 1: 89–104. 11475704

[13] Engelman JA, Mukohara T, Zejnullahu K, Lifshits E, Borras AM, et al (2006) Allelic dilution obscures detection of a biologically significant resistance mutation in EGFR-amplified lung cancer. J Clin Invest 116: 2695–2706. 16906227 10.1172/JCI28656 PMC1570180

[14] Guha M, Castellanos-Rizaldos E, Liu P, Mamon H, Makrigiorgos GM (2012) Differential strand separation at critical temperature: A minimally disruptive enrichment method for low-abundance unknown DNA mutations. Nucleic Acids Res. 10.1093/nar/gks1250 PMC3561944 23258702

[15] Sherry ST, Ward MH, Kholodov M, Baker J, Phan L, et al (2001) dbSNP: the NCBI database of genetic variation. Nucleic Acids Res 29: 308–311. 11125122 10.1093/nar/29.1.308 PMC29783

[16] Milbury CA, Li J, Makrigiorgos GM (2009) COLD-PCR-enhanced high-resolution melting enables rapid and selective identification of low-level unknown mutations. Clin Chem 55: 2130–2143. 19815609 10.1373/clinchem.2009.131029 PMC2828872

[17] Li J, Janne PA, Makrigiorgos GM (2011) Biotinylated probe isolation of targeted gene region improves detection of T790M epidermal growth factor receptor mutation via peptide nucleic acid-enriched real-time PCR. Clin Chem 57: 770–773. 21325655 10.1373/clinchem.2010.157784 PMC3208327

[18] Li J, Wang L, Janne PA, Makrigiorgos GM (2009) Coamplification at lower denaturation temperature-PCR increases mutation-detection selectivity of TaqMan-based real-time PCR. Clin Chem 55: 748–756. 19233916 10.1373/clinchem.2008.113381 PMC2754313

[19] Amicarelli G, Shehi E, Makrigiorgos GM, Adlerstein D (2007) FLAG assay as a novel method for real-time signal generation during PCR: application to detection and genotyping of KRAS codon 12 mutations. Nucleic Acids Res 35: e131. 17932053 10.1093/nar/gkm809 PMC2095824

[20] Li J, Makrigiorgos GM (2009) COLD-PCR: a new platform for highly improved mutation detection in cancer and genetic testing. Biochem Soc Trans 37: 427–432. 19290875 10.1042/BST0370427

[21] Milbury CA, Li J, Makrigiorgos GM (2011) Ice-COLD-PCR enables rapid amplification and robust enrichment for low-abundance unknown DNA mutations. Nucleic Acids Res 39: e2. 20937629 10.1093/nar/gkq899 PMC3017621

[22] Li J, Milbury CA, Li C, Makrigiorgos GM (2009) Two-round coamplification at lower denaturation temperature-PCR (COLD-PCR)-based sanger sequencing identifies a novel spectrum of low-level mutations in lung adenocarcinoma. Hum Mutat 30: 1583–1590. 19760750 10.1002/humu.21112 PMC2784016

