# Supplementary figures and images for: DISSECT Method Using PNA-LNA Clamp Improves Detection of EGFR T790m Mutation
Source: PLoS One. 2013 Jun 21;8(6):e67782. doi: 10.1371/journal.pone.0067782 (PMC3689726; doi:10.1371/journal.pone.0067782)

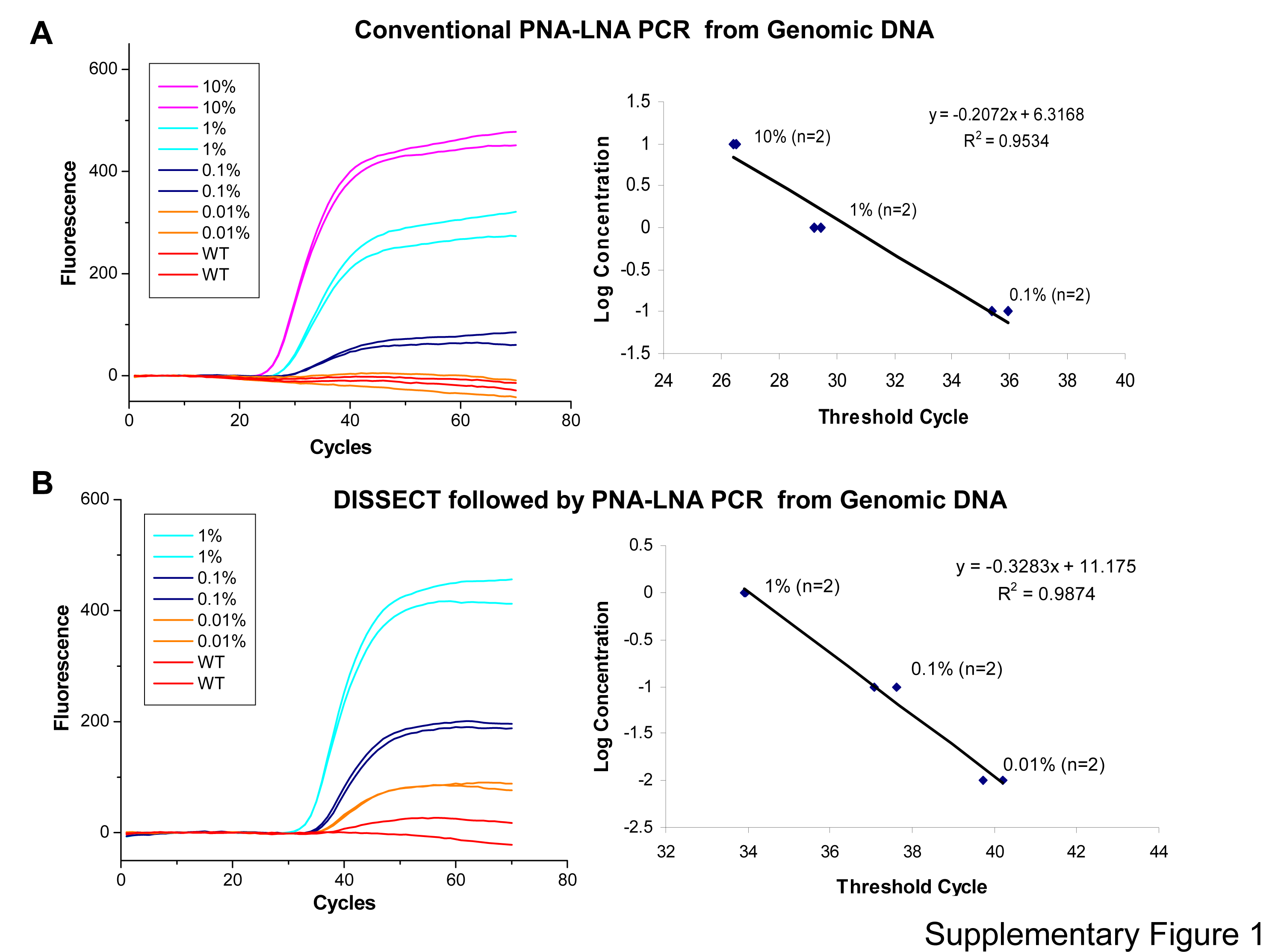

Supplement: Figure S1 — PNA-LNA PCR sensitivity of detection of the T790M mutation when starting from genomic DNA using Phusion Hi-Fidelity™ DNA polymerase. (A) Conventional PNA-LNA PCR showing the detection of the T790M mutant dilutions of 10%, 1%, 0.1% and 0.01% mutant-to-wild type ratios versus wild type alone. (A, right) Corresponding plot of the log concentration of T790M mutant DNA versus threshold cycle number. (B) Real-time PCR plot showing the detection for 1%, 0.1% and 0.01% mutant-to-wild type ratios when PNA-LNA is applied to a sample that has undergone two rounds of mutant enrichment by DISSECT. (B, right) Corresponding graph shows mutant T790M serial dilution following PNA-LNA PCR after DISSECT. (TIF) [file pone.0067782.s001.tif]
